# Electronic monitoring of symptoms and syndromes associated with cancer: methods of a randomized controlled trial SAKK 95/06 E-MOSAIC

**DOI:** 10.1186/1472-684X-11-19

**Published:** 2012-09-24

**Authors:** David Blum, Dieter Koeberle, Karin Ribi, Shu-Fang Hsu Schmitz, Urs Utiger, Dirk Klingbiel, Florian Strasser

**Affiliations:** 1 Oncological Palliative Medicine, Section Oncology, Department of Internal Medicine and Palliative Care Centre, Cantonal Hospital St. Gallen, Rorschacherstrasse 95, St. Gallen 9007, Switzerland; 2 European Palliative Research Center; Dept. Cancer Research and Molecular Medicine, NTNU, Faculty of Medicine, Trondheim, Norway; 3 Oncology, Dept. Internal Medicine and Palliative Centre, Cantonal Hospital St. Gallen, St. Gallen, Switzerland; 4 IBCSG Coordinating Center, Quality of Life Office, Bern, Switzerland; 5 Statistics Unit, SAKK Coordinating Center, Bern, Switzerland; 6 Clinical Trial Management Unit, SAKK Coordinating Center, Bern, Switzerland; 7 Current address: Institute of Mathematical Statistics and Actuarial Science, University of Bern, Bern, Switzerland

## Abstract

**Background:**

In patients with advanced, incurable cancer, anticancer treatment may be used to alleviate cancer-related symptoms, but monitoring of them in daily practice is rarely done. We aim to test the effectiveness of a real-time symptom and syndrome assessment using the E-MO*S*AIC software installed in handheld computer generating a longitudinal monitoring sheet (LoMoS) provided to the oncologists in a phase III setting.

**Methods:**

In this prospective multicentre cluster randomized phase-III trial patients with any incurable solid tumor and having defined cancer related symptoms, who receive new outpatient chemotherapy in palliative intention (expected tumor-size response rate ≤20%) are eligible. Immediately before the weekly visit to oncologists, all patients complete with nurse assistance the E-MO*S*AIC Assessment: Edmonton Symptom Assessment Scale, ≤3 additional symptoms, estimated nutritional intake, body weight, Karnofsky and medications for pain and cachexia. Experienced oncologists will be randomized to receive the LoMoS or not. To minimize contamination, LoMoS are removed from the medical charts after visits. Primary endpoint is the difference in global quality of life (items 29 & 30 of EORTC-QlQ-C30) between baseline and last study visit at week 6, with a 10 point between-arm difference considered to be clinically relevant. 20 clusters (=oncologists) per treatment arm with 4–8 patients each are aimed for to achieve a significance level of 5% and a power of 80% in a mixed model approach. Selected co- variables are included in the model for adjustment. Secondary endpoints include patient-perceived patient-physician communication symptom burden over time, and oncologists’ symptom management performance (predefined thresholds of symptoms compared to oncologists’ pharmacological, diagnostic or counselling actions [structured chart review]).

**Discussion:**

This trial will contribute to the research question, whether structured, longitudinal monitoring of patients’ multidimensional symptoms, indicators for symptom management, and clinical benefit outcomes can influence patients’ quality of life and symptom distress, in a setting of routine oncology practice.

**Trial registration:**

Current Controlled Trials NCT00477919

## Background

In patients with advanced, incurable cancer, anticancer treatment may alleviate patients’ cancer-related symptoms and cancer-associated complications [1]. These beneficial effects may occur even in the absence of a tumor response [2]. In contrast, reduction of tumor size does not necessarily imply a benefit to patients [3]. Chemotherapy may cause physical and psychosocial side effects [4]. An important focus of treatment is therefore to have a beneficial impact on health-related quality of life (HRQL) [5]. HRQL was reported by health care professionals [6] and medical oncologists [7] to be the most important outcome in assessing the effect of palliative chemotherapy. However, HRQL considerations rated by physicians after consultation were poorly associated with decisions regarding modification of palliative chemotherapy [8].

While both monitoring of tumor response and toxicity are defined by gold standards (i.e., RECIST, CTCAE v3.0), symptoms and syndromes, also conceptualized as patient-reported outcomes (PROs), are yet only partially incorporated in routine oncology care [9,10]. Symptoms, which are subjective perceptions of patients, cannot be measured by currently used toxicity scales [11]. Syndromes are mainly clinically described patterns, a combination of symptoms and clinical signs. Cachexia for instance is the combination of the sign weight loss and the symptom anorexia [12].

It is often assumed that an oncologist can estimate the symptoms of the patient accurately using a regular history. However, oncologists’ perceptions may differ from patients’ reported physical and psychosocial experiences. In patients with advanced cancer, the assessment of relevant psychological domains, but also of pain, asthenia/fatigue, or nutritional problems are often underestimated [13,14]. They may not be detected (lack of screening), not be quantified by the patient or by a professional (lack of measuring individuals’ symptom distress) [15,16] or their impact on patients’ everyday functioning is not taken into account (lack of estimation of the magnitude of the problem). Physicians’ concerns about time constraints arising from dealing with unexpected or complex symptoms may contribute to underestimation of symptoms, [17]. In Switzerland, an average of 15 minutes of consultation time is general practice [18].

For the monitoring of anticancer treatment, the palliative effect of chemotherapy on disease-related symptoms and syndromes [15] has been operationalized by defining a clinical benefit criterion. In pancreatic cancer, the endpoint of clinical benefit response (a composite assessment of pain, performance status and weight) was created to provide a way in which the impact of therapy on tumor-related symptoms could be assessed [19] and has become a well-accepted outcome parameter. However, outside of clinical studies its application in routine care is limited. It can therefore be hypothesized, that monitoring of both symptoms, clinical benefit parameters (as objective indicators of effect of management) and selected interventions may result in a quality of life benefit for patients.

There are several approaches pursued to bring patients’ experiences and wishes to the oncology routine care including collection of patients’ symptoms [20], palliative care needs [21], review of systems [22], or general concerns and questions [23], immediately before the visits with physicians and/or nurses. These studies document the proof of concept, that such monitoring can be applied in clinical practice. Looking at the three elements, i.e. symptoms, clinical benefit and treatments, reveals selected documented effects. Monitoring of patients’ symptoms alone increases professionals’ awareness, patient and caregiver’s satisfaction about communication, but rarely effectiveness of symptom management [24]. Monitoring of indicators of patients’ needs, such as declining physical function, distress, repeated hospitalizations, or pre-defined thresholds of symptoms alone will trigger “only” further assessment [14]. Monitoring of current treatments (e.g. pain medications) is only effective, or general concerns and questions [23], immediately before the visits with physicians and/or nurses. These studies document the proof of concept that such monitoring can be applied in clinical practice. Monitoring of patients’ symptoms alone increases professionals’ awareness, patient and caregiver’s satisfaction about communication, but rarely effectiveness of symptom management [24].

The feasibility of self-assessments in patients with advanced, incurable cancer has been demonstrated for various symptom assessment instruments, including the Edmonton Symptom Assessment Scale (ESAS) validated also in cancer outpatient clinics [25].

This study evaluates the effects of the E-MOSAIC intervention, a handheld computer-based assessment of patients’ symptoms, clinical benefit parameters and symptom management information, delivered real-time by the longitudinal monitoring sheet (LoMoS) to oncologists treating patients with anticancer treatment for advanced cancer in palliative intention.

## Methods

This study investigates the effect of E-MOSAIC delivered to oncologists on patient outcomes during a 6 week treatment duration applying a cluster-randomized controlled design.

### Development of intervention tool

Patients with incurable cancer have a high prevalence of symptoms, making it difficult to identify those symptoms essential for routine assessment. Therefore, we decided to select and group the most important symptoms and syndromes into clusters, aiming to maintain an adequate coverage of all important items. This approach should guide the treating physician in a replicable and structured way to monitor relevant (physical and psychological) symptoms and syndromes.

The E-MOSAIC intervention was developed based on pilot work monitoring both symptoms and clinical benefit parameter in oncology care, using paper and pencil to color bars from zero to ten. For symptom assessment, the Edmonton Symptom Assessment Scale (ESAS) is used [26]. The ESAS is a validated nine-item patient-rated symptom visual analogue scale developed for use in assessing the symptoms of patients receiving palliative care. The single item depression of the ESAS can reliably screen for depression as measured by more in depth instrument [27].

From published lists of frequent symptoms the study team selected 21, a number considered both feasible to be utilized in practice and comprehensive enough [28].

**Figure 1:**
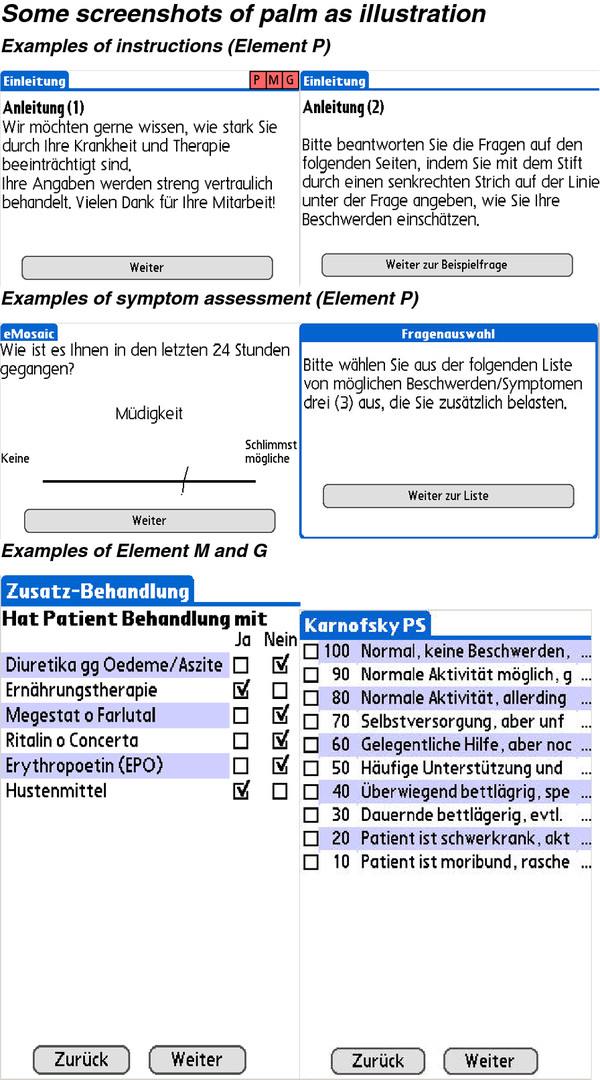
Screenshot from Palm as illustration.

As next step the E-MOSAIC software was developed, piloted and refined with professionals and patients resulting in the palm-based assessment E-MOSAIC (Figure 1).

The palm-based assessment consists of three elements (see screenshots in the Appendix), which are filled out by the patient (element P) and the study personnel (elements G [weight] and M [medication]).

Element P Visual-Analogue Scales (VAS) of

1. Nine frequent symptoms from ESAS (pain, fatigue, drowsiness, nausea, anxiety, depression, shortness of breath, loss of appetite, overall well-being);

For E-MOSAIC the single symptoms of the original ESAS were translated in German, French and Italian language in an informal back- and forward process, and validated preliminarily. ESAS is measured by palm in all patients.

2. Up to three optional symptoms;

3. Patients’ estimated nutritional intake.

Element G:

1. Body weight;

2. Karnofsky Performance Status;

3. Weight loss and body height (Body Mass Index calculated automatically).

Element M: pre-defined, simplified list for actual medication for:

1. Pain syndromes, including assessment of MEDD (Morphin Equivalent [oral] Daily Dose);

2. Fatigue syndromes (Methylphenidate, Erythropoietin, transfusions);

3. Anorexia/cachexia syndromes, and for edema (to control for weight changes).

After completion of the assessments the palm is put back to the docking station and the data are transferred within a few seconds from the docking station to the local computer.

The source-code of the E-MOSAIC software is copy-protected. The software is study-specific, but may be used for other purposes.

**Figure 2:**
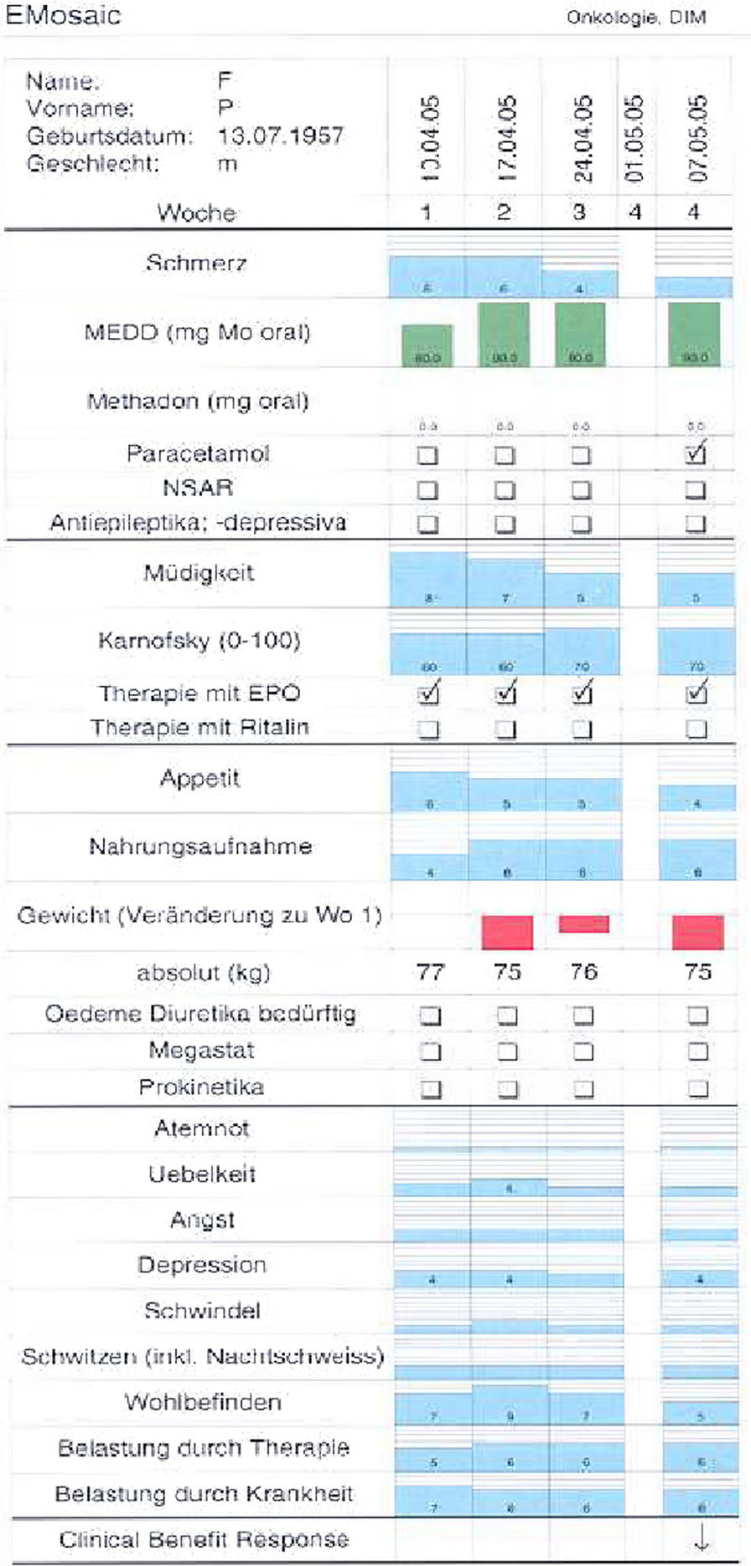
Longitudinal monitoring Sheet: LoMoS.

Longitudinal Monitoring Sheet LoMoS which is printed immediately and put in the patient file for the physicians’ visit by the nurse (Figure 2).

Structure of LoMoS:

1. VAS pain, pain medication (opioids calculated as morphine-equivalent daily dose; other analgesics);

2. VAS fatigue, KPS, medication for fatigue (Methylphenidate, Erythropoietin);

3. VAS anorexia, VAS perceived nutritional intake, weight change, medication for anorexia (nutritional counselling, progestins, prokinetics);

4. 6 ESAS symptoms

5. Maximal 3 of 21 symptoms selected by patient at baseline.

After refining the software, a formal feasibility study was performed as briefly summarized below.

Four centers participated in all or parts of the E-MOSAIC-Feasibility-Study.

Patients filled in the E-MOSAIC in a paper-pen and a palm version in random order.

The compliance, time needed, and experiences of patients with the palm were assessed by a structured 2-page evaluation.

62 patients (median age 64y [30–85], 25 female) participated. 4 patients had visual impairment, 6 comprehension problems, and 1 pat was too tired. 3/62 patients did not complete E-MOSAIC.

Median time to complete was 3 minutes. 10 patients preferred paper and 28 palm, 16 had no preference; 50 patients agreed to continue using palm.

Palm-based symptoms (VAS) were compared with a paper-based categorical symptom assessment (ESAS). Wilcoxon signed-rank tests showed no significant differences between palm and paper of 9 symptoms of element P (p-value: well-being 0.089, dyspnea 0.060, the remaining 7 symptoms 0.249-0.940), but for nutritional intake different (a significant difference was found (.p = 0.013).

Test-retest (1 hour, n = 20) reliability of 9 symptoms and nutritional intake was satisfactory (Cronbach alpha 0.62 - 0.94).

### The E-MOSAIC intervention for this 6-week trial

Although the E-MOSAIC incorporates a module offering the possibility for real-time measurement of clinical benefit response and showing it in the LoMoS the duration of this study of 6 weeks treatment, classical clinical benefit response will not be measured as an outcome, since it needs longer observation to fulfill the criteria.

### Patient population and setting

Patients are eligible who receive anticancer treatment in palliative intention given weekly or biweekly or continuous in the outpatient setting, and routine care which typically includes weekly visits. The setting and routine processes of care include a personal professional nursing contact and a brief patient assessment before the patients visit at the oncologists.

• The palliative intention of the anticancer treatment is defined as an expected tumor response rate ≤ 20% according to literature. To operationalize this definition, a list of tumor types and treatment line was composed (e.g. second line non-small cell lung cancer).

• Patients have to be symptomatic (symptoms measured by VAS: 0 = best, 10 = worst; average over last 24 hours) by the cancer disease, defined as at least one ESAS symptom > = 3/10.

• Patients have to be able to understand the language of the E-MOSAIC assessment and the study related information, written informed consent and the physician is able to communicate with the patient studied without major difficulties (i.e., culture, language, speech).

### Eligible oncologists

Participating oncologists need to be

• experienced in medical oncology and working in a study center or practice, who are likely not to change standard of care for symptom assessment or for major communication skills and who are likely to stay in the participating institution for the time required to treat at least 5 study patients;

• authorized to communicate with the patient about all aspects of cancer care and be authorized to independently perform immediate changes of interventions in patient care without the institutional requirement to counsel another colleague before prescriptions;

• familiar with communication skills, defined as completion of a basic communication skills course or an equivalent training.

### Trial design

**Figure 3:**
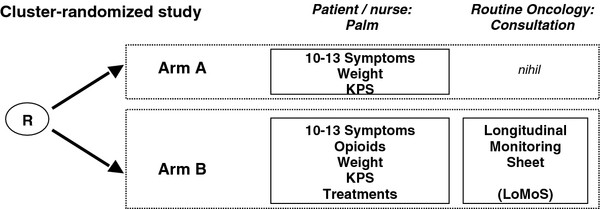
Randomization with intervention.

A cluster-randomized 2-arm design is used to test the E-MO*S*AIC intervention with the LoMoS given to physicians. At enrolment each participating physician will be randomly allocated to one of the 2 arms (standard care, E-MO*S*AIC + LoMoS) at 1:1 ratio stratified according to the institution. All eligible patients to be treated by the physician will be under the same intervention (Figure 3).

After the registration, the palm will recognize the oncologist (scroll bar) and automatically provide the software for the control or the E-MO*S*AIC arm, respectively. After synchronization the unique patient number (UPN) will be updated immediately, with maximal 12 patients per oncologist only 12 patient-UPNs will be possible.

This trial design was chosen in order to minimise contamination. Several patients allocated to the same physicians can hardly be considered independent. In particular, a physician familiar to the LoMoS intervention would probably treat his patients in a similar way, even if they were randomized to different interventions. To prevent this contamination, physicians are chosen as clusters [29]. Cluster randomisation is a standard approach to evaluate both process outcomes and patient outcomes, and is considered especially relevant if the intervention is on physician level and outcomes are patient reported [30].

### Randomization procedure and patient registration

Participating physicians are randomly allocated to the intervention or control arm. Hence, all eligible patients allocated to a physician will be under the same intervention.

Before randomization, the center needs to be activated and the initiation visit has taken place. Each physician has to be informed about the study procedures and has to sign informed consent prior to his randomization. There will be no specific training on symptom management, because the E-MOSAIC intervention in this study includes simply the monitoring sheet. Patient registration is only possible for randomized physicians. Patients give informed consent prior to any protocol-specific procedure.

### Data collection procedures

Patients are seen in all clinics first by oncology nurses who perform the baseline visit, educate patients about the use of the palm, ask patients about oncologist’ interventions in the previous week, and perform at weeks 3 and 6 the outcome assessments.

At baseline, weeks 3 and 6, the cognitive status of patients is assessed.

**Figure 4:**
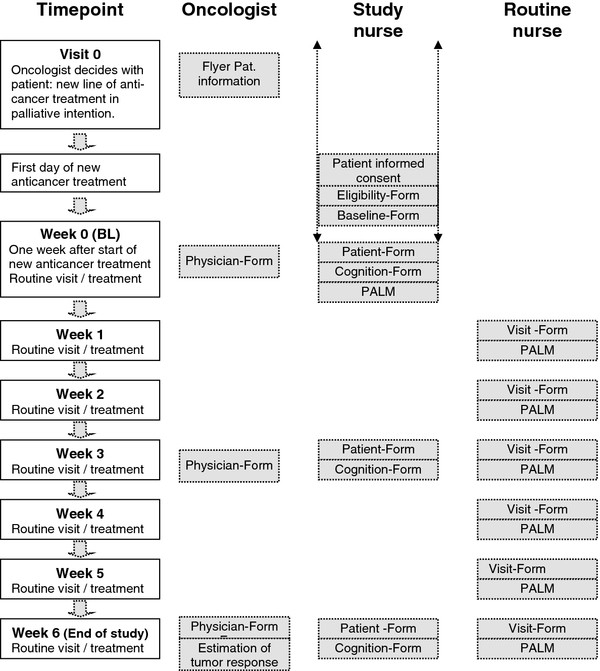
Study flow with visits and forms.

Since mild cognitive impairment is well reported to be underestimated but influencing patients’ ability to express subjective experience of well-being and symptoms, cognitive function will be monitored throughout the study (Figure 4).

To screen for patients with cognitive impairment at baseline, the Mini-Cog, a brief cognitive screening test, will be used. The Mini-Cog and the Mini-Mental Status Examination applied post-hoc to an existing population revealed similar sensitivity (76% vs. 79%) and specificity (89% vs. 88%) for dementia. Therefore, the Mini-Cog test is feasible in settings where time is short, training of personnel is not possible and/or language barriers exist [31].

### Objectives and endpoints

The objective of the study was to evaluate the effects of the intervention using E-MO*S*AIC palm and real-time longitudinal monitoring sheet (LoMoS) in patients receiving anticancer treatment for advanced cancer in palliative intention.

Change in Global Quality of Life (G-QoL) is the primary endpoint. The difference in G-QoL between baseline and after last study visit is measured. The change in QoL will be assessed using the EORTC-QLQ-C30 composed of both multi-item scales and single item measures. Patient will complete the EORTC-QlQ-C30 at baseline and at week 3 and 6 after consultation. G-QoL is the composite score of questions 29 and 30. This instrument is well validated, frequently used and provides a large data base of normative data [32].

Secondary endpoints are the number of patients having a G-QoL response, physician-patient communication, symptoms and syndromes and symptom management performance.

Responders are defined as having a better rated G-QoL assessment after last study visit compared to baseline of more than half of standard deviation of the G-QoL changes of whole study population.

Patients’ estimation of the patient-physician communication will be assessed by a physician compassion rating and general physician attribute rating scales (27).

The rating of the physician compassion uses a semantic differential format including five pairs of physician characteristics. The characteristics are warm-cold, pleasant-unpleasant, compassionate-distant, sensitive-insensitive, caring-uncaring. The two attributes are the left and right anchor of a 100 mm line with each item ranging from 0–100. This scale has been reported to be internally consistent (Cronbach’s alpha coefficient, .92) in cancer survivors and non-cancer patients. A composite score can be calculated (ranging from 0 to 500) [33].

For general physician attributes five other pairs of statements in a semantic differential format will be used: 1) wants best for patients, 2) patient involvement in decision-making, 3) encourage patients’ questions, 4) acknowledging patients’ emotions, and 5) caring for patients. Patients will be asked to rate each of the questions in a scale of 0 (worst) to 100 (best).

Physician’s estimation of the patient-physician communication will be assessed by a questionnaire comprising 7 ad-hoc statements covering satisfaction with amount and clarity of provided information, estimation of patients’ comprehension of information and satisfaction of patient involvement in decision making process. This evaluation tool was developed for the SAKK communication trial [34].

Symptoms and Syndromes from the E-MOSAIC software between baseline and after last study visit will be evaluated. Symptom Distress Score, the summation of the nine ESAS items is used as in the original publication [35]. All ESAS symptoms are individually investigated, to test the hypothesis, that the E-MOSAIC intervention may influence only specific symptoms (e.g., only anxiety and depression, but not pain, or vice versa). As syndromes, pre-defined selected items, the KPS, weight and weight loss, nutritional intake, and use of medical interventions for symptoms (e.g. MEDD) are compared. Several of these items have been conceptualized as clinical benefit criteria.

Number of visits with a symptom load above defined threshold for 5 symptoms without immediate intervention and all interventions performed by the oncologist to alleviate multidimensional suffering of patients and family members will be calculated and oncologists’ routine work will be collected. From the visits all routinely available information describing physician’ diagnostic or therapeutic interventions are collected: visit flow sheets, visit notes and reports, lab sheets, order sheets, and nurses’ notes.

To capture interventions made by the oncologist for any of the multidimensional (symptom) problems, which the patient recalls one week later, the patient will be asked before each visit: “did your doctor prescribe or initiated treatments or interventions to relieve your physical, emotional, or social distress/burden, and if so, please briefly mention them.”

To compare the number of interventions made for key symptoms above defined thresholds. For this study is chosen:

• visits (#) with pain > = 6/10 and no immediate change of analgesics

• visits (#) with fatigue > = 9/10 and no immediate diagnostic or therapeutic intervention

• visits (#) with anorexia > = 9/10 and no immediate diagnostic or therapeutic intervention

• visits (#) with depression > = 6/10 and no immediate diagnostic or therapeutic intervention

• visits (#) with shortness of breath > = 6/10 and no immediate diagnostic or therapeutic intervention

A medical fellow, having at least 2 years clinical experience, who is blinded to the randomisation (the UPI system does not allow to identify institutions nor individual oncologists), will analyse for each patient the visit description and patient recalled interventions of last visit to search for such interventions.

From the visits the following variables are collected: medications and changes, diagnostic interventions, delegated interdisciplinary interventions, and patient perceived interventions.

Additional research questions investigate factors influencing change in G-QoL, tumor response (CR, PR, SD or PD), tumor type, predominant symptom, anxiety, complexity, education and hospitalization. A predominant symptom is defined as the symptom with the highest ranking and all other symptoms are > =2 / 10 lower ranked. Complexity is defined as > =3 symptoms with > =6/10, with the exception of fatigue and anorexia (threshold > =9/10).

To explore patients’ subjective adaptation to illness and burden of treatment two linear analogue self-assessment (LASA) indicators are included, assessing perceived adjustment to chronic illness (PACIS); [34] (‘no effort at all’ – ‘a great deal of effort) and overall treatment burden (‘not at all’ – ’severely’). The indicator for PACIS was confirmed to be responsive to cytotoxic side-effects, mental distress, and psychosocial dysfunction in patients with early breast cancer [36]. It is suitable to describe patients’ adaptation over time. The instruments are validated [37]. The indicator for overall treatment burden has been validated regarding side-effects of antiemetic and cytotoxic therapy [38].

As indicator for decision-making preferences, the difference in number of mismatched decision-making preferences between week 3 and 6 will be compared between the two arms.

Patients’ preferences for involvement in decision making will be assessed by a measure adapted from previous studies [39]. The patient chooses from among five categories ranging from ‘the doctor should make the decision using all that he/she knows about the treatment’ to ‘I should make the decision using all that I know and learn about the treatment’. In addition the physician is asked to choose from among the same five categories how he/she estimates the patients’ preferences.

A mismatch is defined as follows: the patient ranks #1 or #2 and the physician #4 or #5 or vice versa. For neutral patients or physicians no mismatch is possible per definition.

### Sample size calculation

Sample sizes are calculated for an inequality test for two means of change in QoL in a cluster randomized design using the software package NCSS 2004 - PASS 2002, according to the formulation of Donner and Klar, assuming a two-sided significance level of 0.05, and a statistical power of 0.8 [40]. Further assumptions on design parameters are an overall variance (s2) of 400, an intracluster correlation coefficient (ICC, estimated by the ratio of between-cluster variation to overall variance) of 0.05 , an effect size (between-arm difference in G-QoL to be detected) of 10, and the cluster size (the number of evaluable patients per physician) [40]. For the cluster size several options are considered, but it is expected to stop the trial at a cluster size of 8 with 12 physicians per arm, yielding a total sample size of 192 evaluable patients.

Since the initial estimate of the ICC might not be appropriate, an interim analysis to adjust the sample size as suggested in Lake et al. is foreseen [41]. Once data for the first 100 patients are available, estimates of within-cluster variation and between-cluster variation are obtained. If the resulting ICC has to be at least 1.5 times larger than the value taken for the initial sample size estimation, then the sample size would be adjusted using the new estimate of ICC, without alteration on other design parameters. The feasibility of patient accrual is evaluated at the same time. The recommended cluster size might be adjusted accordingly.

### Data analysis

Due to the cluster structure, comparisons of different outcomes between treatment arms will be analyzed by mixed models. For endpoints with continuous values, linear mixed model may be applied. For endpoints with categorical or binary values, nonlinear mixed model or generalized estimating equations may be applied.

The data will be stored and analyzed at the SAKK Coordinating Center using SAS software, Version 9.2 of the SAS System for Windows (SAS Institute Inc., Cary, NC, USA) and the open source R statistical software package (http://www.r-project.org/). All statistical tests will be done two-sided at a significance level of 0.05. P-values will be corrected for multiple testing where appropriate.

Descriptive statistics will be done by median and range for continuous variables. Categorical data will be reported using absolute and relative frequencies.

For the *primary endpoint*, selected influential variables (education, tumor type, predominant symptom, anxiety, complexity, hospitalisations) and the baseline G-QoL value will be included in the analysis model as covariates. For the primary analysis, only evaluable patients will be used. As a sensitivity analysis, non-evaluable patients will be included if possible. For instance, the difference between baseline and 3 weeks will be analyzed including patients who are evaluable at week 3 but non-evaluable at week 6.

Several pre-defined subgroup analyses are foreseen: The difference in G-QoL will be compared between both arms in sub-groups of patients having

a) a tumor size response (SD, PR, CR) or not (PD),

b) basic education or additional education

c) one of the main tumor types defined as composing > = 20% of the evaluable study patients.

d) a predominant symptom, if composing > = 20% of the evaluable study patients (expected based on symptom epidemiology data: pain, anorexia and/or fatigue [both predominant vs. other symptoms or alone vs. other symptoms], anxiety and/or depression [both predominant vs. other symptoms or alone vs. other symptoms], nausea, shortness of breath).

e) anxiety <6/10 or > =6/10

f) complexity less than 3 symptoms above threshold vs. > = 3 symptoms above threshold (fatigue and anorexia > =9/10, other symptoms > =6/10).

All subgroup analyses will include baseline G-QoL as covariate.

The study population will be described separately by institution (study center)-, oncologist-, and patient-related factors.

The study center will be described with regards to actual procedures of symptom and syndrome assessment at the participating institution and local available interventions for multidimensional symptom and syndrome management.

From the participating oncologists following data are collected: Gender, age, mother language, living > =6 months in the language region and Board certification oncology.

The patients included will be characterized with respect to socio-demographic variables, cognition, and disease and treatment-related variables.

### Ethical considerations

This protocol was written, and the study is to be performed in accordance with the Declaration of Helsinki and the Guidelines of Good Clinical Practice issued by ICH. The study has been approved by the local ethics committees of the cantons of all participating centers (Aarau, Basel, Bern, Zuerich, Fribourg, Graubuenden, St.Gallen, Ticino). There is no approval outstanding.

All patients are informed of the aims and procedures of the study. They are informed as to the strict confidentiality of their data, but they need to know that their medical records may be reviewed for study purposes by authorized individuals other than their treating physician.

Informed consent is obtained on a written form approved by the local ethics committee. Two copies of the informed consent have to be signed, one of which is handed to the patient.

Patients have the right to refuse further investigations for any reason and at any time. Patients who decide to withdraw from the study should be asked whether they also want to withdraw their consent for their data to be used for the follow-up assessments. It is emphasized that participation is voluntary and that the physician is allowed to refuse further participation in the study whenever he/she wants. Physician’s informed consent is obtained on a written form.

## Discussion

This study evaluates the effects of longitudinal assessment of symptoms and syndromes in patients receiving anticancer treatment for advanced cancer in palliative intention on health related quality of life, symptoms, communication and physicians performance.

### Interim analysis

The interim analysis was based on the data from 89 patients coming from 31 different physicians. Of those, the data from 8 patients could not be used, since they were assigned to physicians who enrolled only one patient, i.e. they cannot be used to estimate within cluster correlation. At that time already 160 patients allocated to 47 physicians had been accrued. Unexpectedly a negative ICC was found. A literature search on negative ICCs only revealed that this appears in case of very similar but rather small clusters [42]. This translates to a negligible between-cluster variation and a dominant within-cluster variation which in turn is the case for very small ICCs. Then a simulation study using the design parameters of this trial and a SAS macro for power calculations (fpower.sas) was performed [43]. It seemed that an increasingly negative ICC does not diminish the power, but rather that the power increases further. Hence the sample size was not adapted for this reason.

However, during the interim analysis three problems arose regarding the sample size:

a) None of the predefined scenarios met the reality of cluster sizes accumulated in the first three years: instead, the vast majority of clusters were smaller than expected.

b) An unexpectedly high number of patients were not evaluable for primary endpoint according to the requirements specified in and implied by the protocol. Although the original protocol focused on evaluable patients, it did not foresee additional patients to be accrued to account for non-evaluable patients.

c) There were many clusters with only one or two or even no patients. These are problematic because they do not allow to properly calculating the within-cluster correlation required for the interim and final analysis. In fact, this renders patients who are evaluable of limited value for the analysis. While the trial design allows for varying and even small cluster sizes it is not clear how these very small clusters will affect the final results. Moreover, while there are approximate formulae to adjust the final sample size for varying cluster sizes, these only apply to approximate calculations which were not used during the trial development. These problems revealed from the interim analysis led to the following conclusions which were documented in a note to file:

1) 160 patients will be required, distributed to four patients per cluster with twenty clusters per treatment arm.

2) An additional five per cent, i.e. eight patients, will be accrued to account for varying cluster sizes.

3) That is, in total 168 evaluable patients will be accrued to meet the design specifications of the protocol (alpha = 5%, power = 80%).

Because of the problems described in b) and c) even 240 accrued patients (the maximum foreseen in the protocol although there only evaluable patients were mentioned) would not have been sufficient to obtain 168 evaluable patients. Hence, it was decided by the trial team members at the SAKK CC on April 20, 2011 to accrue an additional 10 percent, i.e. 24 patients, resulting in a total of 264 patients.

### Context

Several studies assessed whether the provision of HRQL data to oncologists, using touch pad symptom assessment devices, [24,44] by using prompt sheets [45,46] or summaries of HRQL, [47] improve communication between oncologist and patient and symptom control. The provision of a summary of HRQL (EORTC-QLQ-C30) to patients and oncologists in a randomized crossover trial resulted in more frequent discussion of HRQL issues and detection of unexpected psychosocial topics and symptoms [14].

Longitudinal symptom assessment by means of computers is feasible in the multicenter setting even for a long time span. In a recently published study patients were invited to report symptoms (Common Terminology Criteria for Adverse Events CTCAE) on an online platform. Of 125 invited, 105 participated for the mean length of one year and showed a high compliance and high satisfaction with the system, however there was only a marginal effect on communication [11].

Randomized controlled trials focused mainly on improvement of communication and symptom distress. A palm based interactive tailored patient assessment, containing a selection of symptoms, problems and concerns, rating of this items from 1–4 and prioritization for support was tested in 145 lymphoma and leukemia patients in a single centre in Norway. The assessment output was immediately delivered to treating physicians and nurses in the intervention group. The outcomes measured were the same assessments: Numbers of symptom problems and concerns addressed, change in symptom distress and need for support. In the intervention group there were more symptoms addressed, less symptom distress measured and patient were less in need for symptom management support [48].

A second randomized controlled trial investigated the effect of the ESRA-C (electronic self-report assessment cancer) on in 660 cancer patients at two institutions. The output from the ESRA-C was displayed to the treatment team in the intervention group. The primary outcome was the likelihood of discussion of symptoms and quality of life issues (SQLIs) between clinicians and patients and the secondary endpoints the visit duration and the perceived usefulness by clinicians. When the SQLIs were considered as problematic, they were more frequently discussed during the visit in the intervention group; the length of visit time was equal between the two groups. The clinicians perceived the output as useful [20].

### Strengths

The strength of our approach, is the defined clinical setting, chemotherapy in palliative intention, where disease related symptoms, treatment related toxicities and clinical benefit parameters guide treatment.

In contrast to the previous studies which aimed at a general improvement of communication our study aims to improve symptom control due to more in depth symptom assessment and adaption of chemotherapy due to better monitoring of toxicity and clinical benefit parameters.

A second strength is the multicenter setting which tests the intervention in a real life environment and differences between centers can be further studied.

The main focus on specific and generic patient reported outcomes (quality of life, and symptoms) reflects patient centered care in the oncological setting.

### Limitations

E-MO*S*AIC intervention may be effective on several levels (i.e., awareness of patient, awareness of physician, coping, symptom control, communication), this study tests the hypothesis to overall improve the quality of palliative cancer care, rather than focusing on specific outcomes only. Global single-item QoL indicators are similarly efficient as multi-item scales for overall treatment comparisons and changes over time because they reflect the summation of the individual meaning and importance of various factors [38]. The use of a single-item tool to appropriately obtain a measure of overall QoL was reported from a cooperative multicenter study setting [49].

Patient-rated QoL may be influenced by many factors. Disease and treatment-related as well as social and cultural factors are essential for any individuals’ estimation or judgment of their QoL. Therefore we correct for known influential factors in the analysis.

The differences between the randomised physicians may have a bigger influence than the intervention, especially on communication. Patient-physician communication can affect the psychological distress and quality of life of cancer patients [50]. Informativeness, interpersonal sensitivity, and partnership building, three dimensions of communication, are related to patient satisfaction, compliance, and medical information recall [51]. Several elements of the patient-physician communication have been emphasized, such as recognition of patients’ main concerns related to physical but also emotional dimensions, fulfilling the patients’ individual and general information needs, [52-54] a physician-communication style reflecting empathy, care, compassion, and understanding of patient’s difficulties to cope, [55] physicians’ ability to break bad news [56,57] and recognition of patients decision-making preferences [58].

The management of common symptoms is part of oncologists’ professional skills. The Global Core Curriculum for Medical Oncology (ASCO/ESMO) includes supportive and palliative care items and the Quality Cancer Care statement of ASCO and ESMO includes pain management, supportive and palliative care [59].

A large body of evidence builds the foundation for practice guidelines in symptom management of advanced cancer patients, allowing agreed-on classifications of various types of symptom control interventions and thresholds of symptom expression. However, as in other medical disciplines, variability in symptom management practice is common, driving academic exchanges including research and scholar literature. Therefore, in this study, no practice guidelines are provided nor explored.

The reactions to symptoms may therefore vary between physicians as well as the documentation of the interventions.

### Future

The E-MO*S*AIC study carries the potential to improve certain aspects of clinical management in daily oncology practice for patients with advanced, incurable cancer by simple, real-time, longitudinal monitoring of patient-reported outcomes. The intervention tested is a step towards the development of longitudinal clinical benefit outcome strategies for disease-oriented trials.

A well-accepted and feasible instrument to document patient-reported outcomes may improve the use of anti-neoplastic treatments in patients with advanced cancer.

As a potentially relevant spin-off of the E-MO*S*AIC study, the interdisciplinary collaboration of oncology nurses and oncologists may be fostered. The competencies of oncology nurses in patient care may become better acknowledged.

## Abbreviations

ASCO: American society of clinical oncology; CRF: Case report forms; DOB: Date of birth; EC: Ethics committee; E-MOSAIC: Electronic monitoring of symptoms and syndromes associated with advanced cancer; ESAS: Edmonton symptom assessment scale; ESMO: European society for medical oncology; FACT: Functional assessment cancer therapy survey; GI: Gastro intestinal; G-QoL: Global quality of life; HRQL: Health-related quality of life; ICH: International conference on harmonization; KPS: Karnofsky performance status; LASA: Linear analogue self assessment; LoMoS: Longitudinal monitoring sheet; MEDD: Morphin equivalent daily dose; MSAS: Memorial symptom assessment scale; NSCLC: Non small cell lung cancer; PACIS: Perceived adjustment to chronic illness scale; PRO: Patient-reported outcomes; QoL: Quality of life; SMF: Study master file; SCLC: Small cell lung cancer; UPI: Unique physician identifier; UPN: Unique patient number; VAS: Visual-analogue scales; WHO: World Health Organization.

## Competing interests

The authors declare that they have no competing interests.

## Authors’ contributions

BD, KD, RK, HSS-F, UU, KD, SF made substantial contributions to conception and design, or acquisition of data, or analysis and interpretation of data; BD, KD, RK, KD, SF have been involved in drafting the manuscript or revising it critically for important intellectual content; and BD, KD, RK, HSS-F, UU, KD, SF have given final approval of the version to be published.

## Pre-publication history

The pre-publication history for this paper can be accessed here:

http://www.biomedcentral.com/1472-684X/11/19/prepub
